# Recording of Falls in Elderly Fallers in Northern Greece and Evaluation of Aging Health-Related Factors and Environmental Safety Associated with Falls: A Cross-Sectional Study

**DOI:** 10.1155/2022/9292673

**Published:** 2022-01-07

**Authors:** Dimitrios Lytras, Evaggelos Sykaras, Paris Iakovidis, Konstantinos Kasimis, Ioannis Myrogiannis, Anastasios Kottaras

**Affiliations:** ^1^Department of Physical Education and Sports Sciences, Aristotle University of Thessaloniki, 57001 Thermi Thessaloniki, Greece; ^2^Department of Physiotherapy, Faculty of Health Sciences International Hellenic University-Alexander Campus, P.O. Box 141, 57 400 Sindos, Thessaloniki, Greece; ^3^Medical School, Faculty of Health Sciences, Aristotle University of Thessaloniki, GR-54124 Thessaloniki, Greece

## Abstract

**Background:**

Elderly falls constitute a global problem with huge social and economic aspects. Fall risk factors are both intrinsic (physical and psychological) and extrinsic (related with environmental safety).

**Aim:**

To record both intrinsic and extrinsic risk factors and their correlation in elderly fallers in order to suggest specific guidelines for their medical care and environmental modification inside and outside the home.

**Method:**

The study involved 150 elderly fallers (median age 70 (67-74)), who completed a record containing information on known risk factors related to their health status, as well as information on the conditions and causes that led to the fall. Each fall was considered an independent event, while measurements were performed regarding balance, strength, their functional ability, and the fear of a possible fall. Descriptive analysis and frequency analysis were used to record the health and activity status of the participants as well as the fall-related environmental factors. Severity of each fall event across a variety of locations was examined using the Kruskal-Wallis one-way analysis of variance. Multiple linear regression was applied to examine the effect of the mean values of functional tests and medical records on the number of fall events.

**Results:**

In the span of 12 months, a total of 304 fall events were recorded. Regarding location, 77.6% occurred indoors; more frequent were the bedroom (28.6%) and the bathroom (28%). The interior stairs (10.5%), the kitchen (4.9%), and the living room (3.3%) were the less frequent locations. Concerning danger, falling on the interior stairs caused the longest hospitalization, followed by the kitchen and the bathroom. Extrinsic factors that led to both indoor and outdoor falls were the administration of psychotropic medication, poor space ergonomics, lack of basic safety standards, and poor lighting conditions. Vision problems and dizziness resulted in more falls than other intrinsic factors. Furthermore, reduced performance in the FICSIT-4 test and the 30-Second Chair Stand Test, as well as high scores in the CONFbal–GREEK questionnaire and the Short FES-I, shows a linear relationship with an increased number of falls.

**Conclusions:**

Ergonomic interventions can help prevent indoor elderly falls. Poor construction and lack of adequate lighting mainly cause outdoor falls. Regular eye examinations, management of vertigo, improvement of the balance and strength of the lower limbs, and reduction of fear of impending falls are the intrinsic factors that help prevent falls the most.

## 1. Introduction

Falls are a major concern for older adults. Statistics reveal that people over the age of 65 are 30% more likely to have at least one fall per year, while the rate rises to 50% for older adults over 80 [1, 2]. Furthermore, country-specific records show that falls accompanied by injury are responsible for 70,000 hip fractures per year being the leading cause of mortality of traumatic etiology in older adults in the UK [3], whereas in the USA, more than 3 million people visited hospital emergency departments due to fall-related injuries and more than 1.6 million were eventually hospitalized [4] Furthermore, falls, in addition to immediately deteriorating the health of older adults, are accompanied by psychological side effects, as they negatively impact the confidence of older adults, increase the tendency for isolation, and reduce their ability to self-care [1, 5–7].

Regarding the economic aspect of falls, the USA was impacted with $23.3 billion in 2008 and $5 billion in 2010 in related health costs, [4] while the UK is burdened with £1.7 billion annually [3].

The causes of falls are multifactorial and are related both to the effects of aging on the human body and to environmental factors. Recent fall prevention guidelines [1–3] state that specific health problems that occur within older adults such as polypharmacy, loss of balance, and impaired sensory function (vision and hearing) are at fault for an increased risk of falls. Additionally, the environment of older adults seems to be an important factor leading to falls, as it often contains various risks such as slippery floors, poor ergonomics of spaces, or insufficient lighting [3, 8]. The combination of the above factors along with the lack of information and education of older adults about falls seems to play a decisive role in the number of falls that occur annually [2, 9, 10].

In Greece, data on the recording of falls in older adults is limited. In a study by the Accident Research and Prevention Center of the University of Athens, in the period 1996-2003, approximately 30.694 falls were recorded from 1996 to 2003. In terms of causality, stumbling and slipping are the major causes (70%); gender-related statistics show that women are more prone to fall (72%) and, regarding the area, falls occur primarily in the residence (house or nursing home). As far as injurious falls are concerned, half of all falls result in a fracture. Hospitalization is required for up to 44% of older adults aged 85 or older (hospitalized for up to two weeks) and for 16% of those in the age bracket 65-74 (average stay in hospital is 9 days). Finally, mortality was recorded at 90 losses [11].

Recent research data have shown that the main goal when dealing with falls in older adults is to identify populations which are high-risk (such people over the age of 65), so as to perform appropriate interventions and create suitable guidelines for fall prevention [12–14]. Furthermore, people who have already experienced a fall are referred to as “fallers” and are the primary focus of this research, because they are at a much higher risk to experience a recurrent severely injurious fall [3, 12, 13, 15]. Multifactorial interventions related to the secondary prevention of falls have been shown to be able to reduce the chances of a new fall by up to 50% [16]. These interventions include exercise, education, and information for older adults as well as modifications of their environment in order to remove the risks that can lead to a fall [12, 17–20].

Although falls seem to be a major health problem in Greece, to date, no attempt has been made to record the episodes, nor any targeted intervention to prevent them in terms of changes in the environment of older adults [21, 22]. The aim of this study is to capture the true dimension of the problem of falls in older adults and to try to identify the related factors. More specifically, the purpose was to record falls (with or without injury) and to assess both intrinsic (history, concomitant health problems) and extrinsic (pills, slippery floors, poor lighting, ergonomics, etc.) risk factors associated with them in order to draw useful conclusions and to develop appropriate strategies for their prevention, both in terms of instructions for regular medical care and the implementation of appropriate home modifications.

## 2. Method

This was a cross-sectional study conducted under the supervision of the Department of Physical Education and Sports Science in Thessaloniki. It involved 150 older adult fallers, members of a total of 15 Open Care Centers for the Elderly (KAPI) located in five different cities of Central Macedonia in Greece. Ethical approval was granted by the Ethics Committee of the Aristotle University of Thessaloniki (No. 44338/2020).

### 2.1. Procedure

Registered members of 15 Open Care Centers for the Elderly (KAPI) from five different cities in northern Greece were invited to participate in this research from December 2019 to February 2020. The invitations were sent by the physiotherapists employed in the KAPI, with the consent of their board of directors. Members were informed about the research by local press releases, on-location posters, printed invitations, and telephone. Finally, informative speeches were held, where specialized physiotherapists expanded on the dangers of falls in older adults and attending members were asked to declare their interest in participation in the research. The informative talk included the description of the risk factors for falls, ergonomic safety rules inside and outside the home, and the training of older adults in matters of fall management.

Any person who was interested in participating was then booked an appointment on-site with the researchers to check whether they met the inclusion criteria or not. If they met the criteria, they were provided with relevant information leaflets and were asked to provide their written consent.

At the next meeting, the participants of the research were asked to fill in an anonymous measurement sheet, which contained data regarding their demographic characteristics, their activity status, their social background, and their state of health. Each participant was asked to recall the fall episodes they had in the last 12 months and for each of them to fill in a fall record, which included data about environment factors during the fall incident. Each fall was considered an independent event, and each participant was asked to complete as many records as the number of falls they had experienced during the last 12 months. Where possible and after the consent of the participants, the content of the fall records was verified in terms of their accuracy and correctness through communication (by phone or live) of members of the research team with relatives of each participant.

### 2.2. Participants

The study involved 150 older adult fallers aged 65-80 years (median age 70 (67-74)), who were screened for eligibility and recruited over a three-month period (from December 2019 to February 2020). To be eligible, patients had to be men and women aged 65-80 with a history of at least one fall in the last 12 months, be ambulatory, and have a score in the Timed Up-and-Go (TUG) test of less than 15 seconds [23]. Exclusion criteria were a diagnosis with neurodegenerative disease (e.g., Parkinson's disease), recent stroke, and senile dementia (Mini-Mental State Exam score less than 24) [24].

### 2.3. Therapists

This research included three physiotherapists specialized in geriatric physiotherapy (10.2-year average experience working with older adults).

### 2.4. Outcomes

#### 2.4.1. Measurement Sheets and Fall Record

The following sociodemographic characteristics were collected through a measurement sheet: gender, age, level of physical activity, social background, and general health status (medical history) (Table 1). From all the factors related to their state of health as well as their lifestyle, those were selected whose presence has been associated with an increased risk of falls in advanced age [3, 25]. Additionally, a fill-in form was used to record each fall (Table 2), which contained multiple choice questions related to the incidence of each fall (e.g., whether the fall was traumatic or not, part and time of day of the fall, cause, lighting conditions, and shoe suitability) and also asked the participant to give a brief description of each fall in terms of the conditions that led to it.

#### 2.4.2. Mini-Mental State Exam (MMSE)

The tool used to assess the cognitive impairment of the participants was the Greek version of MMSE (http://repository.edulll.gr/edulll/bitstream/10795/2173/23/2173_07_Mini-Mental-State-Examination.pdf), and the cut-off values were based on the instructions of Fountoulakis [24]; a score lower than 22/30 was considered valid for diagnosing dementia. To assess the balance, the strength of the functional level of the participants, and the fear associated with falls, the following outcomes were measured by the same blind assessor.

#### 2.4.3. Timed Up-and-Go (TUG) Test

An appropriate tool to assess mobility and evaluate balance in older adults is the TUG test [26, 27], because it has been shown to have high intrarater and interrater reliability in that age group [26]. The following steps are required to complete the test: (1) rise from a sitting position on a chair, (2) walk normally to a marked spot on the floor (3 meters), (3) turn around, (4) walk the distance in the opposite direction, and (5) resume the sitting position on the chair. The evaluation point is the time required for the subject to complete the sequence of movements (https://www.cdc.gov/steadi/pdf/TUG_Test-print.pdf). Podsiadlo and Richardson [27] state that a time of 20 seconds or less indicates the ability of the individual to function independently inside and outside their home. Mean scores divided by age groups are as follows: 65-69 years 8.1 seconds (7.1–9.0 CI), 70-79 years 9.2 seconds (8.2–10.2 CI), and 80-99 years 11.3 seconds (10.0-12.7 CI) [23]. In order to achieve functional homogeneity of the participants, the cut-off point in this study was set at 15 seconds.

#### 2.4.4. Frailty and Injuries: Cooperative Studies of Intervention Techniques (FICSIT-4) Balance Test

This tool is used to evaluate the static balance of a person. It consists of seven tests of increasing difficulty, with a score range of 0-28. Thus, each test is individually scored from 0 (requires assistance to prevent falling) to 4 (fully able to stand for 10 seconds independently). The examinee performs four stances and then repeats three of them with closed eyes (thus, seven tests in total). These stances are standing with their feet side by side, placing the instep of one foot so that it is touching the big toe of the other foot, tandem stance (heel-to-toe), and standing on one foot. The last one is performed only with open eyes (http://geriatricphysio.yolasite.com/resources/geriatric_assessment_tool_kit.pdf). Rossiter-Fornoff et al. [28] tested the test-retest reliability of the FICSIT-4 balance test to 187 older adults from the community twice in a period of 3-4 months and found moderate interclass (Pearson) correlations (0.66).

#### 2.4.5. 30-Second Chair Stand Test

To identify high-risk older adults, the Centers for Disease Control and Prevention created the Stop Elderly Accidents, Deaths, and Injuries (STEADI) toolkit [29], a part of which is the 30-Second Chair Stand Test. This tool is used to evaluate leg endurance and strength in older adults. The participant, after crossing their arms at the wrists against their chest, repetitively sits and stands from a chair for 30 seconds. The evaluation point is the number of times the individual is able to perform this action. The five- or 10-repetition sit-to-stand tests were impaired by the floor effect, which this test has overcome. The 30-Second Chair Stand Test has also exhibited excellent test-retest reliability in a total number of participants: *r* = 0.89 (95% confidence interval 0.79-0.93) [30].

#### 2.4.6. Berg Balance Scale (BBS)

Berg et al. proposed the BBS as a tool to evaluate balance in older adults [31, 32]. The scoring of the 14 tests of the BBS is similar to that of the FICSIT-4 test, that is, 0 (unable to perform the task) to 4 (able to perform the task unassisted). The tests are of gradual difficulty, and the subject is asked to either perform a certain task or hold a stance for a given time. Berg et al. [32] state that a score of 45 or less shows fall-related balance impairments, while functional balance is indicated with a score of 56. Furthermore, the BBS has exhibited high intrarater and interrater reliability with intraclass correlation in the range of 0.98-0.88 in older adults [33].

#### 2.4.7. CONFbal–GREEK Questionnaire

Regarding the assessment of balance confidence during daily activities, a suitable tool is the CONFbal scale questionnaire, with its Greek version (CONFbal–GREEK) being used in this study [34]. It consists of 10 questions revolving around the performance of daily skills and their associated confidence. The score of each question ranges from 1 (confident in performing the task) to 3 (not confident at all). Therefore, to exhibit confidence, an individual needs a lower score. According to Simpson et al. [35], the questionnaire demonstrates excellent internal consistency (Cronbach's alpha 0.91, with an intraclass correlation coefficient of 0.95) and excellent test–retest reliability.

#### 2.4.8. Short Falls Efficacy Scale International (Short FES-I) Score Questionnaire

Concerning the psychological aspect of falls, a tool to measure the related fear or concerns is the Falls Efficacy Scale International (FES-I). The Greek version [34] of its short form (Short FES-I) was used in this study, containing seven questions in total. The score ranges from 7 to 28. Each answer is scored from 1 (not concerned at all) to 4 (extremely concerned); thus, a low score indicates a low fear of falling. According to Kempen et al. [36], the test has shown excellent internal and 4-week test-retest reliability (Cronbach's alpha 0.92, intraclass coefficient 0.83).

### 2.5. Statistical Analysis

Data were analyzed using SPSS Statistics for Windows, version 25.0 (SPSS Inc., Chicago, IL, USA). *Ν*ormal distribution was checked with the Shapiro-Wilk test as well as with the appropriate graphs (Q-Q plots and P-P plots). Normally distributed variables were presented with mean value and standard deviation or with median value and interquartile range in the case of abnormally distributed variables. Respectively, the quality variables were presented with frequencies and percentages. The dependent variables that we examined were indicative of the severity of a fall (days of hospitalization after an injurious fall event) and of the proneness of the fallers to accumulate fall events (number of fall events in the last year). Regarding our independent variables, we examined the severity of each fall event across a variety of locations (both in domestic and outside environments) using Kruskal-Wallis one-way analysis of variance (since the homogeneity of variance assumption of one-way ANOVA was violated). The susceptibility to engaging in a fall event was examined relative to the faller scores on the tests mentioned previously and, also, relative to their medical records. We used multiple linear regression to examine the effect of each covariate on the number of fall events.

## 3. Results

In the given time frame (December 2019-February 2020), the 15 KAPI managed to recruit 224 older adults for the research. After being checked for meeting the inclusion criteria, the study eventually involved 150 individuals. Table 1 presents their demographic characteristics, as well as their health and activity status.

From the total number of participants, a total of 304 fall events were recorded, which took place over a period of 12 months. The characteristics of the falls as recorded from the questionnaires are summarized in Table 2.

According to the recording of fall events, the vast majority of the number of falls occurred in the house with the bedroom and bathroom showing the highest incidence of fall events compared to other areas of the house (interior stairs, kitchen, and living room). A large proportion of falls (over 60%) occurred at night (either before bedtime or during bedtime, when the fallers got up to go to the bathroom or kitchen for water). A large percentage (about 40%) of older adults stated that poor lighting conditions or vision problems played a very important role in the fall, while a much less important causal factor seems to have been the inappropriate shoe or the fact that they were barefoot.

Regarding the manner of fall, the participants reported that they most often slipped or tripped on an object. In the bedroom area, falls occurred during the night. According to older adults, what led to the fall was to stumble either on bedcovers that were lying on the floor or on an object that they had left on the floor next to their bed or on a cable from a telephone or electrical device (heater, lamp) and even to slip on a slippery floor or on a rug next to the bed. Most of these falls occurred in the dark, and often, older adults reported that they did not have easy access to light or a small lamp next to their bed.

In the bathroom, most of the falls according to the description of the events took place when the person entered or left the shower (or bathtub). In the vast majority, even after a fall, there was no safety measure such as the installation of support handles and nonslip floors inside and outside the shower, while there were limited cases where there was a special seat inside the bathtub. Less common in the bathroom were falls when sitting and getting up from the toilet. To be noted is that even after these falls, no protection measures were taken even, such as installation of support handles on the wall or toilet lift.

In the interior stairs, most of the falls, according to the descriptions of older adults, occurred due to abandoned objects on the stairs or on the staircase, in combination with poor lighting conditions, while less often, the fall was due to slippery stairs or inappropriate shoes. In most cases, the basic ergonomic safety rules were not observed such as nonslip mats on the stairs, handrails on both sides, adequate lighting, and easy access to a light switch at the bottom and top of the stairs. Even after a traumatic fall on the indoor stairs, older adults did not enter the process of making the above modifications, either because they did not know what to do or because they were not receptive to modifications in their home.

In the kitchen area, most falls occurred when older adults in their attempt to reach something from above climbed on a stool or chair and lost their balance. Falls in the attempt to move food from the kitchen to the dining room or living room were also frequent. Many falls were also due to a slippery carpet in front of the sink or oils or grease on the floor combined with an unsuitable shoe. Similar to previous areas, even after the incident of a fall, neither the individuals themselves nor their relatives made ergonomic modifications in the kitchen area.

The living room had the fewest falls compared to any other area of the house. The falls usually occurred when older adults stumbled on an object lying on the floor or on a cable or carpet, while less often, the fall was due to a slippery wooden floor combined with an unsuitable shoe.

Finally, a very small number of falls inside the house (*n* = 7) occurred in places that we had not included in the record of falls such as boiler room, garage, and storage room.

In terms of outdoor falls, they covered only 22.4% of all falls with the largest percentage of them occurring on the balcony or in the yard. The main reason for these falls was to slip due to wet floor in combination with inappropriate footwear, while the fall often occurred in poor lighting conditions. Concerning falls in other outdoor areas, they covered a very small percentage of the total number of falls (Table 2) and occurred in areas such as the road, the park, the sidewalk, and the outdoor stairs. It was noteworthy that in these falls in a fairly large percentage, it was reported that the fall was caused by some defect of the outdoor area, (asphalt potholes, broken sidewalks, worn support railings, and nonworking lamps).

**Figure 1:**
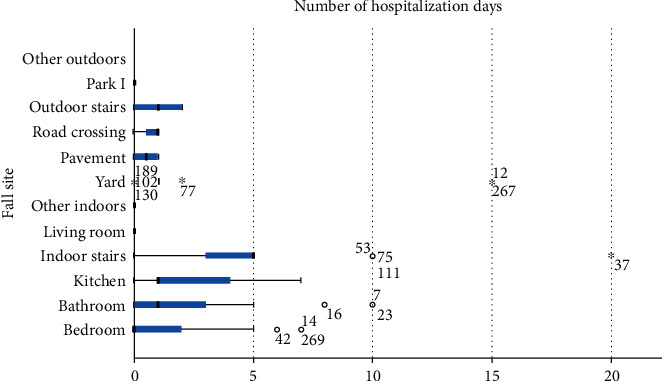
Box and whisker plot of the number of hospitalization days for each fall site.

To examine the differences among the different locations that the injurious fall events occurred, we selected only the fall events that resulted in injury (203 injurious fall events out of 304 total). Levene's test (Levene statistic = 2.543, *p* = 0.007) showed that the variances of our dependent variable (hospitalization days) among the different locations were not equal and we used the Kruskal-Wallis *H* test. The test showed that there was a statistically significant difference between the fall locations regarding the hospitalization time (*H* = 41.09, *p* < 0.001).  Table 3 and Figure 1 present the mean number of days of hospitalization for each fall location. The higher number of hospitalization days was observed when using indoor stairs, followed by the kitchen and the bathroom. All these fall sites are locations of everyday use; hence, the need to adopt ergonomic interventions in such locations is high.

Using multiple regression to test the effect of each one of the Timed Up-and-Go, 4-Stage Balance, 30-Second Chair Stand, Berg Balance Scale, CONFbal–GREEK questionnaire, and Short FES-I score tests on the number of fall events, we found a statistically significant predictive power of our six covariates as a whole (*F*(4, 143) = 42.90, *p* < 0.001, *R*^2^ = 0.643). Specifically, the statistically significant (*p* < 0.001) covariates of our model were four: (a) the FICSIT-4 test and the 30-Second Chair Stand Test which reduce the number of falls as their scores increase and (b) the CONFbal–GREEK questionnaire and the Short FES-I score which increase the number of falls as their scores increase.  Table 4 summarizes the regression outcome for the effect of each one of the functional tests performed.

Multiple linear regression was also performed to assess the effect of a variety of qualitative variables from the medical record of the fallers. The model was found to have predictive power (*F* = 23.42, *p* < 0.001, *R*^2^ = 0.691). Of the thirteen covariates from the medical records, three were found to have a statistically significant effect (*p* < 0.001) on the number of falls: the administration of psychotropic pills, the impairment in vision, and vertigo events. Specifically, patients prescribed for psychotropic pills, patients with vision impairment, and patients experiencing vertigos were in increased risk of experiencing a fall event.  Table 5 summarizes the regression outcome for the effect of each one of the medical record variables.

## 4. Discussion

The aim of this study was to record the characteristics of falls in older adult fallers in a wider area of northern Greece (Central Macedonia), to identify the main causative factors that can lead to an increased risk of traumatic falls, and to suggest guidelines for a more appropriate behavior on health issues, as well as appropriate modifications of the environment of older adults to avoid falls.

Based on their profile (mean age and physical activity status), the participants in this study were relatively active seniors. However, the results of the functional test measurements (Table 1) revealed deficits in both strength and balance as well as in their functional ability, a fact which is confirmed by their mean values, which were found to be lower than those of healthy people in the respective age groups [37, 38]. For instance, the mean TUG values of the participants, when taking into account their mean age and fall event history, were similar to the values of older adults at mild risk of experiencing a fall [23]. Likewise, the mean value of the 30-Second Chair Stand Test score was low compared to the normal values (12-13 lifts) proposed by Jones et al. [39, 40]. This seems mostly normal, since the participants in our study were fallers, so it is logical for them to show those characteristic deficits associated with the physiological effect of aging on the human body (intrinsic fall risk factors) [12, 41].

Multiple regression analysis revealed that the good performance in the FICSIT-4 balance test and in the 30-Second Chair Stand Test seems to have affected more the number of falls compared to the performance in the other functional tests (Table 4). The fact that the FICSIT-4 test evaluates the static balance, while the 30-Second Chair Stand the endurance and strength of the lower limbs, led us to the conclusion that perhaps interventions aimed at improving these specific parameters may be more effective in reducing falls than others in older fallers. Hopewell et al. [2] state that the key to multifactorial interventions is targeted exercise and, more specifically, exercise programs for secondary fall prevention, such as the Otago exercise program [19]. Exercise has been shown to be the most important treatment in preventing falls in older adults [1, 5, 16, 42].

It is noteworthy that these deficits of the participants in functional ability, strength and balance, do not seem to be affected by the fact that some of the participants exercised. Therefore, we concluded that simple walking, dancing, and cycling can contribute to maintaining good health and well-being but do not appear to be sufficient to compensate for deficits in static and dynamic balance, as well as in strength in the static muscles of the lower limbs. This conclusion is in line with the NICE guidelines [3], according to which simple exercise interventions, such as dancing and walking, do not seem to have a significant effect on the secondary prevention of falls. In contrast, exercise programs for secondary fall prevention such as the Otago exercise program, which include a combination of resistance and balance exercises mainly on the lower limbs, according to research can reduce falls by up to 50% [1, 2, 13, 16, 43, 44]. It is, therefore, imperative that multifactorial interventions for the prevention of falls include the organization and implementation of secondary prevention exercise programs in older adults.

Another important finding of the results in our research was the strong effect we found of the score of the CONFbal–GREEK questionnaire and the Short FES-I on the total number of falls (Table 4). This result highlights in our opinion the importance of the effect of psychological aspects of falls on older adults, such as fear of impending fall and reduced balance confidence. It is possible that the increased fear of falling will lead to avoidance of activity and further deterioration of the functional level of older adults. The results of our research confirm the significant impact of the psychological side effects of falls on the functional ability of the elderly as they negatively impact the confidence of older adults, increase the tendency for isolation, and reduce their ability to self-care [1, 5–7]. Thereby, we believe that interventions for secondary fall prevention should include information and training on fall management and prevention in order to reduce the fear and insecurity of older adults.

Regarding the medical history of the participants, from the health problems that were reported, those that seemed to affect the increased number of falls the most were the vision problems, the taking of psychotropic drugs, and the episodes of vertigo (Table 5). These medical factors have also been identified by NICE as significant risk factors for a possible fall [3]. It is worth noting that while a relatively large percentage of participants stated that they have vision impairments (30%), only 34% underwent eye examinations in the last two years. It is always advisable for health professionals involved in the care of older adults to check their history for the presence of these risk factors while it is important to encourage them to have regular eye examinations.

As for the education of older adults about fall prevention issues, we were very impressed by the fact that even if they had experienced one or more fall incidents, the vast majority did not know basic fall protection rules. This became apparent during the informative talks that took place at the premises of KAPI, in which we provided educational material on ergonomic interventions in the home as well as educational videos on how older adults can avoid or even deal with a fall inside their home. We were particularly impressed that a vast majority of seniors had no prior information about home modifications, as all participants had experienced one or more falls and in fact, 77% of all falls had occurred indoors. Even in cases of people who had one or more falls in certain areas of the house, they did not make any modification of these areas, mainly because they were not informed and less often because they did not want to; i.e., they were not receptive to changes in their home area.

This was a very important finding of our research, as we identified a significant deficit in the information and education of older people around this issue. Recent research has shown that informing and educating older adults are an integral part of multifactorial interventions in fall prevention programs in older adults [1, 2, 10]. Therefore, in the future, more informative speeches should be conducted, adapted for older adults, concerning ergonomic interventions in the home area.

### 4.1. Suggestions for Ergonomic Interventions Indoors and Outdoors to Prevent Falls

Regarding the safety of the spaces inside the house, based on the description of the fall events of the participants and how they could be avoided in the future, we have to suggest the following ergonomic interventions.

In the bedroom area, it is very important that there are no objects left on the floor next to the bed (books, medicine boxes) and also no electrical or telephone cables. It is good to have a nonslip mat with a thick pile which protects the individual in case of a fall. Special care is also needed in bed linen which may be touching the floor. However, the most important thing is the easy access to the light on the nightstand as well as the placement of night light that stays on all night.

In the bathroom, it is necessary to place special support handles on the wall at the entrance and exit of the shower (or bathtub), as well as the installation of a special seat in the shower. Special nonslip floors must also be installed inside and outside the shower. In addition, special support handles should be placed in the toilet area, while in patients with orthopedic problems, the use of a toilet lift is recommended.

In the kitchen area, special attention should be paid to the floor in front of the kitchen hobs and the sink, where it is good to place a nonslip carpet. The arrangement of the items in the kitchen cabinets should be such that the items used daily are low and thus, the person avoids going up somewhere to reach something from above. The use of an ergonomic special ladder with a large support base, which has nonslip stairs and a wheeled table for transporting food, can significantly reduce the risks in the kitchen area.

It is very important that there are no objects left on the stairs or on the stairwell. It is also necessary to install nonslip tape or carpet on the stairs. There must be a handrail, ideally on both sides, and adequate lighting with easy access to the switch (e.g., installing an “aller retour” switch at the top and bottom of the staircase).

In the living room or dining room, it is very important that there are no objects left on the floor, as well as cables that vertically cross the living room floor. If there is a wooden floor, it is recommended to install nonslip carpets or to place double-sided tape on carpets that are slippery.

Particularly important of all the above modifications are those of the interior staircase and less of the kitchen and bathroom, as they are associated with an increased risk of injury and hospitalization duration.

Regarding the safety of the outdoor areas, according to the findings of this study, the most dangerous outdoor area for injury and increased hospitalization compared to other outdoor areas is the balcony or the yard. It is recommended that older adults be especially careful in their yards and balconies, especially when the floor is wet. Installing nonslip tiles or special nonslip floors can reduce the risk of a fall. It is also suggested to avoid outdoor work at night, while it is beneficial to have adequate lighting outside the house.

Many of the above suggestions are in line with suggestions from other researchers regarding modifications of the environment inside [45–47] and outside the home [48, 49]. The adoption of appropriate home modification according to Chase et al. [8] has been shown to drastically reduce the number of falls.

### 4.2. Precautions and Behaviors That Should Be Adopted by Older Adults Themselves to Prevent Falls

Based on the findings of this research, it is recommended that older adults who have dizziness in their medical history, those who take psychotropic drugs, and those who have vision problems be especially careful. They should have regular eye examinations and use the right eyeglasses. Furthermore, special care should be taken in choosing the appropriate footwear, which should have a nonslip sole, and they should avoid walking barefoot in the house. The above suggestions are in line with the results of other studies [50, 51], which also highlight the importance of vision disorders and dizziness as health factors for causing falls.

Finally, a key factor for the protection of older adults is to inform and educate them about issues related to falls. They should seek to be informed and to consult specialists in order to proceed with the appropriate interventions inside and outside the home. On the other hand, the state should create appropriate structures and control bodies that could assess the safety of the homes of older adults, provide them with relevant information, and suggest ergonomic interventions.

### 4.3. Limitations

The main limitation of this research was the fact that it coincided with the beginning of the COVID-19 pandemic in Greece and the closure of KAPI for a long time. The initial design of the study included the participation of other cities in Central Macedonia and a larger number of participants. However, this was not possible as the closure of KAPI forced us to stop the recruitment of participants and to limit it to the five cities of the prefecture.

## 5. Conclusion

The vast majority of falls in older adults in Central Macedonia occur indoors. The main finding of our research was the complete lack of education of older adults on the prevention of falls, both in terms of postures and health behaviors and in terms of ergonomic interventions in their home. Many of the falls that occurred indoors could have been avoided with appropriate ergonomic interventions, while the falls outside seem to be caused more by construction defects and lack of basic safety standards in outdoor public areas. Furthermore, the regulation of psychotropic drugs is also advised. Regarding the intrinsic factors of falls, the regular examination of the eyes and the management of vertigo seem to have a significant effect on the prevention of new episodes of falls. Improving the balance and strength of the lower limbs and reducing the fear of impending fall through appropriate exercise and information/education interventions for older adults seem to contribute more to reducing the number of falls compared to other factors.

## Figures and Tables

**Table 1:** Demographic characteristics and medical history of participants.

Age (years)	70 (67-74)
Gender (female)	88.7% (*n* = 133)
Do you live alone? (yes or no)	21.3% (*n* = 32) yes
Are you in charge of the care of your spouse or any other relative at home? (yes or no)	19.3% (*n* = 29) yes
Do you have regular blood check-ups? (yes or no)	82% (*n* = 123) yes
Have you checked your eyes in the last two years? (yes or no)	34% (*n* = 51) yes
Number of falls during the last year	2 (1-2.75)
Number of pills per day	2 (1-2)
Do you make regular use of sedatives or antidepressants? (yes or no)	17.3% (*n* = 26) yes
Eyesight problems (yes or no)	30% (*n* = 45) yes
Problem with your blood pressure (hypertension or hypotension) (yes or no)	15,3% (*n* = 23) yes
Osteoporosis (yes or no)	22.7% (*n* = 34) yes
Incontinence (yes or no)	14% (*n* = 21) yes
Pacemaker (yes or no)	0.7% (*n* = 1) yes
Gait and posture instability (poor balance) (yes or no)	44.7 (*n* = 36) yes
Musculoskeletal problems that make it difficult to walk (e.g., osteoarthritis of the hip or knee) (yes or no)	20% (*n* = 30) yes
Vertigo (yes or no)	11.3% (*n* = 17) yes
Heart problems (yes or no)	7.3% (*n* = 11) yes
Diabetes (yes or no)	13.3% (*n* = 20)
Other health issues (yes or no)	6% (*n* = 9) yes
*Time of physical activity per week (hours/week)*	
None	35.3% (*n* = 53)
1-2 hours per week	50.7% (*n* = 41)
3-5 hours per week	13.3% (*n* = 20)
More than 5 hours per week	0.7% (*n* = 1)
*Type of physical activity*	
None	37.3% (*n* = 56)
Walking or running	38.7% (*n* = 58)
Dancing	14.0% (*n* = 21)
Biking	8% (*n* = 12)
Working out	2% (*n* = 3)
Other	0% (*n* = 0)
Mini-Mental State Exam score^∗^	28 (26-29)
TUG sec (SD)	11.43 (1.40)
FICSIT-4 test sec (SD)	21.07 (3.96)
30-Second Chair Stand Test times (SD)	9.99 (2.53)
BBS	45.57 (5.09)
CONFbal–GREEK score (SD)	13.57 (2.69)
Short FES-I score (SD)	12.56 (3.3)

^∗^Score range 23-30 (indicates a normal cognition). A higher score on the test means a lower risk of dementia.

**Table 2:** Characteristics of falls.

Were you seriously injured in the fall? (yes or no)	66.8% (*n* = 203) yes
Days of hospitalization	1.33 (2.59)
Fall location (indoors/outdoors)	77.6% (*n* = 236) indoors
Area of the house where the fall occurred	
Bedroom	28.6% (*n* = 87)
Bathroom	28% (*n* = 36)
Indoor stairs	10.5% (*n* = 32)
Kitchen	4.9% (*n* = 15)
Living room	3.3% (*n* = 10)
Other	2.3% (*n* = 7)
Outdoor area where the fall occurred	
Yard or balcony	10.2% (*n* = 31)
Crossing the road	5.9% (*n* = 18)
Park	3% (*n* = 9)
Sidewalk	2% (*n* = 6)
Stairs	0.7% (*n* = 2)
Other	0.7% (*n* = 2)
How did the fall occur?	
Slipped	43.1% (*n* = 131)
Tripped on something	33.6% (*n* = 102)
Felt dizzy	9.5% (*n* = 29)
Lost balance while sitting up or down	8.2% (*n* = 25)
Trying to reach something high	3.9% (*n* = 12)
Other	1.6% (*n* = 5)
To what extent was the fall due to improper footwear or the fact that you were barefoot?	
Very much	10.2% (*n* = 31)
Much	10.2% (*n* = 31)
Moderately	3.6% (*n* = 11)
A little	28.9% (*n* = 88)
Not at all	45.7% (*n* = 139)
To what extent was the fall due to inadequate lighting or poor vision?	
Very much	39.5 (*n* = 120)
Much	12.8 (*n* = 39)
Moderately	2.6 (*n* = 8)
A little	8.6 (*n* = 26)
Not at all	36.5 (*n* = 111)
What time of day did the fall happen?	
Morning	5.3% (*n* = 16)
During the day	34.9% (*n* = 106)
Night	29.9% (*n* = 91)
At bedtime	29.9% (*n* = 91)

**Table 3:** Mean days of hospitalization for each location of the fall events.

Fall site	Days of hospitalization	
	Mean	Standard deviation
Bedroom	1.11	1.87
Bathroom	1.96	2.43
Kitchen	2.30	2.36
Indoor stairs	4.52	4.07
Living room	0.00	0.00
Other (indoors)	0.00	0.00
Yard	2.63	4.86
Sidewalk	0.50	0.71
Road crossing	0.75	0.50
Outdoor stairs	1.00	1.41
Park	0.00	0.00
Other (outdoors)	1.99	2.96

**Table 4:** Model coefficients of each functional test affecting the number of fall events.

Covariate	*β*	s.e.	C.I. low	C.I. up	*p*
Timed Up-and-Go	0.016	0.039	-0.062	0.093	0.692
FICSIT-4	-0.053	0.014	-0.080	-0.025	<0.001
30-Second Chair Stand	-0.120	0.026	-0.170	-0.069	<0.001
Berg Balance Scale	0.004	0.011	-0.018	0.026	0.703
CONFbal-Greek questionnaire	0.109	0.025	0.060	0.158	<0.001
Short FES-I	0.092	0.022	0.049	0.135	<0.001

**Table 5:** Model coefficients of each medical record variable affecting the number of fall events.

Covariate	*β* (as difference of “yes” from “no” answers)	s.e.	C.I. low	C.I. up	*p*
Psych. pills	+1.54	0.22	1.11	1.97	<0.001
Eye check-up	-0.14	0.11	-0.35	0.08	0.211
Blood test	+0.05	0.13	-0.22	0.31	0.714
Vision impair.	+0.45	0.12	0.22	0.71	<0.001
Incontinence	-0.02	0.21	-0.45	0.40	0.912
Hypertension	-0.22	0.14	-0.50	0.07	0.132
Osteoporosis	+0.14	0.12	-0.11	0.38	0.265
Pacemaker	+0.38	0.63	-0.87	1.63	0.549
Balance diff.	+0.05	0.11	-0.17	0.27	0.667
Knee-hip	+0.11	0.13	-0.15	0.36	0.402
Vertigo	+0.68	0.18	0.32	1.04	<0.001
Cardio	+0.39	0.20	-0.02	0.78	0.062
Diabetes	+0.26	0.16	-0.05	0.57	0.094

## Data Availability

The authors confirm that the data supporting the findings of this study are available within the article.
